# Primary Reapproximation of a Near-Amputated Distal Fingertip With Nail Bed Involvement and Minimal Soft Tissue Attachment: A Case Report

**DOI:** 10.7759/cureus.112702

**Published:** 2026-07-15

**Authors:** Oscar A Vazquez, Taryn Gilgallon, Kevin Abadi, Anupam Gupta

**Affiliations:** 1 Surgery, University of Florida, Gainesville, USA; 2 Surgery, Herbert Wertheim College of Medicine, Miami, USA; 3 Emergency Medicine, Florida Atlantic University, Boca Raton, USA; 4 Surgery, Jackson Memorial Hospital, Miami, USA; 5 Surgery, Bangalore Medical College, Bangalore, IND

**Keywords:** hand, reconstruction, skin graft, surgery, trauma

## Abstract

Minimally attached near-complete distal fingertip amputations are often treated with revision amputation due to concerns of vascular compromise. Composite graft survival in adults has long been viewed as unpredictable, especially in smokers. Survival rates vary widely, and smoking has been identified as an independent risk factor for graft failure. We describe an 18-month follow-up of an Allen type II near-amputated distal fingertip (nail bed involvement without bone exposure), with an approximately 3-mm volar dermal bridge. The injury was managed with primary reapproximation using interrupted nylon sutures and a transungual nail plate suture for stabilization. Estimated ischemia time was approximately 2.5 hours. Despite chronic nicotine exposure, the patient achieved complete epithelialization, normal nail growth, preserved 4-mm static two-point discrimination, excellent cosmesis, and full functional recovery at 18 months. Only mild persistent subjective numbness compared to the contralateral fingertip was reported. This case demonstrates that when perfusion remains evident and the mechanism is a sharp cutting injury, primary salvage can succeed even with minimal attachment and a smoking history.

## Introduction

Fingertip injuries distal to the distal interphalangeal (DIP) joint without bone exposure are usually managed nonoperatively because of the rich vascular supply to the pulp [1]. Near-complete amputations with only tenuous soft tissue attachment, however, create a therapeutic dilemma. Many surgeons opt for revision amputation out of concern for necrosis rather than attempting salvage with full-thickness skin grafts from the palm. The Allen classification categorizes fingertip amputations into four types based on the level of tissue loss. Type I involves only the pulp distal to the nail bed. Type II involves the nail bed without bone exposure. Type III involves the nail bed with partial loss of the distal phalanx. Type IV extends proximal to the lunula and involves the germinal matrix [2,3].

Outcomes after distal replantation and composite grafting in adults show variable survival rates that depend on the mechanism of injury, level of amputation, and patient factors such as smoking. Reported survival rates range from 43% to 93% [4,5]. Recent evidence has identified the mechanism of injury as a critical determinant of composite graft survival. Sharp cutting injuries demonstrate significantly higher success rates compared with crush or avulsion injuries [6,7]. In a study of 94 patients undergoing composite grafting, multivariate analysis revealed that crushing injury was independently associated with graft failure. Cutting injuries, grafting within five hours of injury, and nonsmoking status were associated with favorable outcomes [6].

The distal pulp has a dense subdermal vascular plexus fed by the terminal digital arteries. This provides enough redundancy to allow revascularization even when attachment is minimal [8]. Smoking has been linked to impaired microvascular function and higher risk of graft failure in several series [4,9], though the magnitude of this effect remains debated. Some studies have found that mild to moderate cigarette consumption of 20 cigarettes per day or fewer did not significantly increase the risk of replant failure. Only heavy smoking of more than 20 cigarettes per day was identified as a significant risk factor [10]. Nail bed injuries also demand accurate realignment to avoid permanent deformity [11].

We present durable 18-month survival and functional recovery after primary reapproximation of a near-amputated distal fingertip (Allen type II) attached by only a 3-mm dermal bridge in a chronic nicotine user following a sharp cutting injury.

## Case presentation

A 27-year-old right-hand-dominant female presented approximately 40 minutes after a sharp knife laceration while cleaning dishes. She reported chronic nicotine use through vaping (about one vape containing 50 mg of nicotine consumed every two weeks). On examination, there was a near-complete amputation of the tip of her right middle digit distal to the DIP joint. The distal segment remained attached by an approximately 3-mm volar dermal bridge, as determined by visual intraoperative estimation. The nail plate and nail bed were disrupted without exposed bone or tendon, consistent with an Allen type II injury [2]. The wound base showed active bleeding, and capillary refill in the distal segment was brisk, suggesting preserved perfusion. Sensation was grossly intact upon presentation. Radiographs showed no distal phalanx fracture.

Since vascularity appeared preserved, the mechanism was a sharp cutting injury, and there was no crush component or skeletal involvement, primary salvage was chosen. Local anesthesia was achieved with 3 cc of 1% lidocaine without epinephrine by local infiltration. This approach was selected given the tenuous soft tissue attachment and the surgeon’s preference to avoid any potential vasoconstrictive effect in a marginally perfused segment.

**Figure 1:**
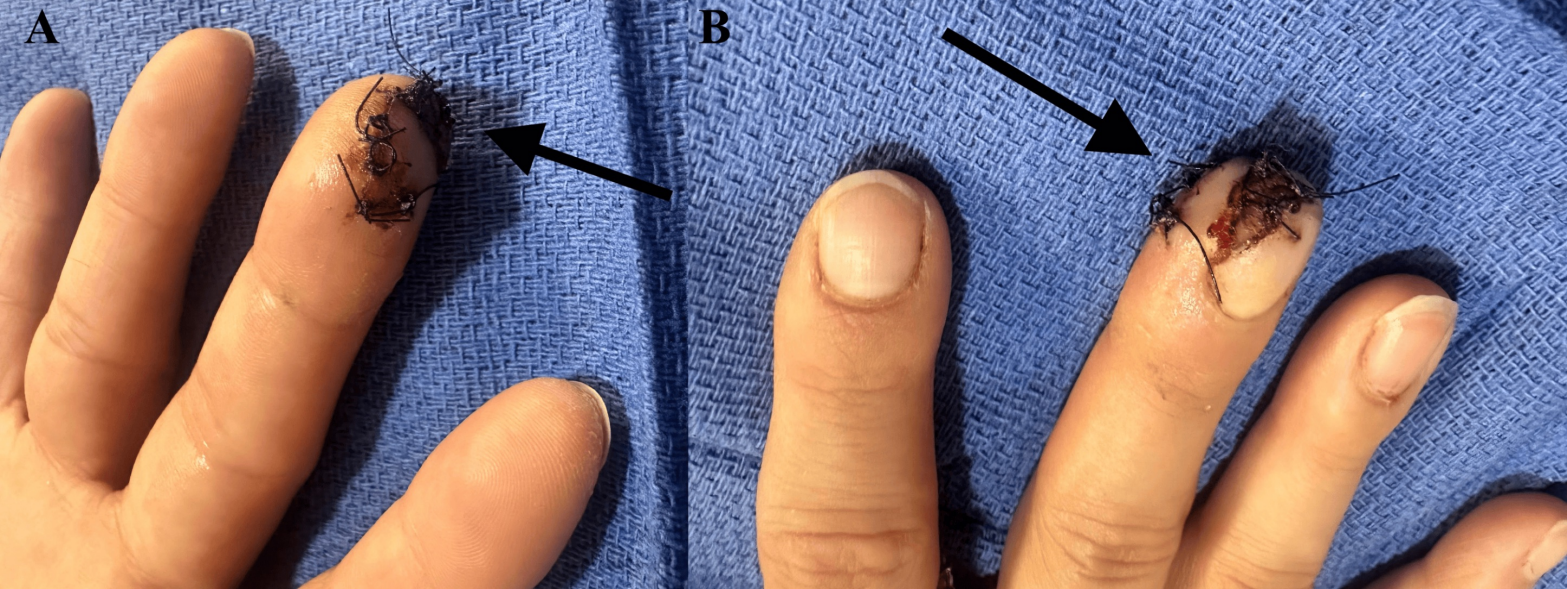
(A) Immediate postoperative appearance (volar view) showing reapproximated distal fingertip with interrupted 4-0 nylon sutures. (B) Immediate postoperative appearance (dorsal view) showing the reapproximated distal fingertip with interrupted 4-0 nylon sutures and transungual nail plate stabilization suture.

Copious saline irrigation and minimal debridement were carried out. The distal segment was reapproximated with interrupted 4-0 nylon sutures, taking care to avoid tension across the dermal bridge (Figure 1A). A 4-0 nylon suture was passed transungually through the nail plate to stabilize the nail bed and maintain alignment (Figure 1B). The wound was dressed with Xeroform, sterile gauze, and micropore tape. No splint was used. Prophylactic antibiotics were not given because the wound appeared clean and perfusion was preserved. The estimated time from injury to repair was about two and a half hours.

**Figure 2:**
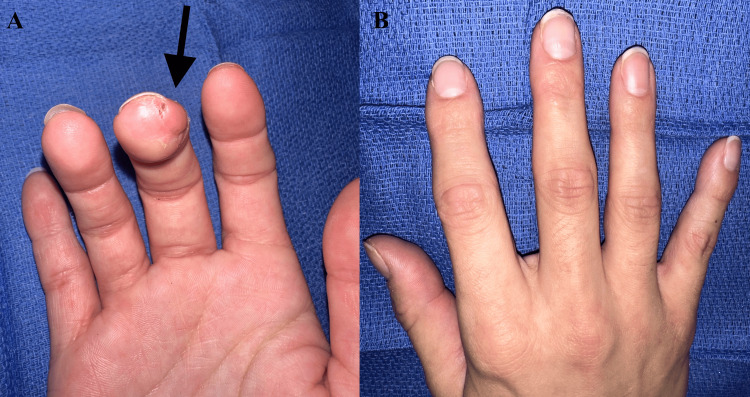
(A) Two-month follow-up (volar view) demonstrating complete epithelialization and preserved pulp contour with small apical deformity. (B) Two-month follow-up (dorsal view) demonstrating complete nail growth.

The patient continued nicotine use during the postoperative period. At two weeks, the sutures were removed, and the fingertip showed intact soft tissue coverage with early epithelialization and preserved contour at the volar and dorsal aspects. At two months, complete epithelialization had occurred with normal nail growth (Figure 2). Static two-point discrimination measured 4 mm, which is within the normal range of 2 to 5 mm for the fingertip pulp [12,13]. The patient denied hypersensitivity or cold intolerance but noted mild subjective decreased sensation compared to the contralateral fingertip. Capillary refill remained brisk.

**Figure 3:**
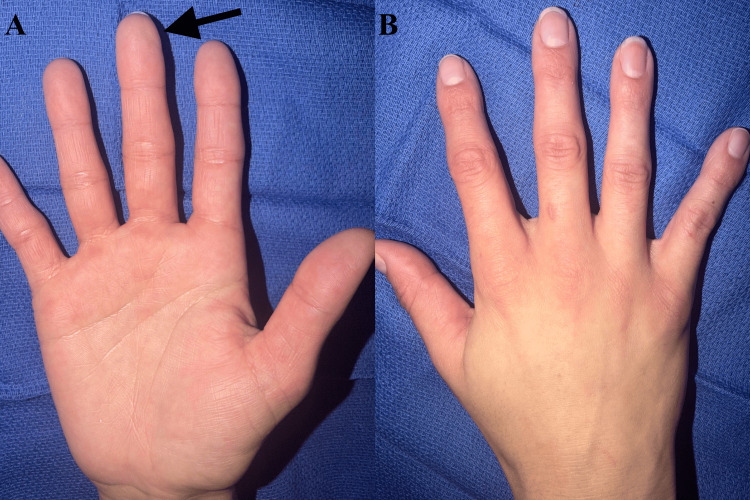
(A) Eighteen-month follow-up (volar view) showing preserved fingertip length, symmetric pulp volume, and normal skin texture compared with adjacent digits. (B) Eighteen-month follow-up (dorsal view) demonstrating normal nail growth without hook deformity, ridging, or dystrophy.

At 18 months, the fingertip retained preserved length, symmetric pulp volume, and normal contour relative to the opposite side (Figure 3). Nail growth remained normal without hook deformity, ridging, or dystrophy. Static two-point discrimination remained 4 mm. The patient still reported mild subjective hypoesthesia compared to the contralateral side but denied hypersensitivity, cold intolerance, or functional limitation. The cosmetic result was excellent, and no secondary procedures were needed.

## Discussion

Distal fingertip injuries benefit from robust vascularity, which often allows for good outcomes even when attachment appears tenuous [1]. Composite graft survival and distal replantation results in adults vary, with lower survival rates than in children and outcomes affected by mechanism, ischemia time, and patient factors [5]. The mechanism of injury has emerged as one of the most important predictors of success. In a study of 211 fingertip replantations, crush or avulsion injuries were associated with a 41% failure rate compared with significantly lower failure rates in sharp-cut injuries. Logistic regression identified injury mechanism as a statistically significant predictive factor [7]. Similarly, in Tamai zones I-II replantations, sharp-cut injuries demonstrated a 91.7% survival rate versus only 46.2% for crush-degloving injuries [14].

The role of smoking in composite graft and replantation outcomes remains nuanced. Heistein and Cook identified smoking as the only significant factor with a strong, independent association with graft loss in their prospective study of 57 digital tip amputations [4]. However, other studies have reported more variable findings. Zhu et al. found that only heavy cigarette consumption of more than 20 cigarettes per day significantly increased the risk of replant failure, while mild and moderate consumption did not [10]. He et al. found that smoking did not significantly affect replantation outcomes in their cohort of 149 replants, though crush and avulsion injuries significantly increased failure risk [15]. These findings suggest that while smoking remains a concern, it may not be an absolute contraindication to salvage attempts, particularly when other favorable factors are present.

Although this injury retained a minimal dermal bridge and therefore is not a true composite graft, the tenuous attachment shares similar physiology. Early survival would be expected to rely on plasmatic imbibition in the first 24 to 48 hours, followed by inosculation and progressive neovascularization across the preserved dermal bridge. Brisk capillary refill and active bleeding at presentation strongly suggested sufficient arterial inflow to support the process. The dense arterial plexus of the distal pulp can facilitate revascularization even with minimal soft tissue attachment [8]. In this case, several factors likely contributed to successful integration despite chronic nicotine exposure. First, the sharp-cutting mechanism without a crush component preserves vessel integrity and minimizes the zone of injury [6,7]. Second, perfusion was preserved at presentation with brisk capillary refill. Third, the short ischemia time of approximately 2.5 hours was well within the five-hour window associated with favorable outcomes [6]. Fourth, the patient was young. Fifth, minimal debridement preserved maximal tissue. Sixth, an epinephrine-containing anesthetic was used according to the surgeon's preference.

Precise nail bed alignment is essential to prevent long-term deformity [11]. Stabilization with a transungual nylon suture gave adequate support during early healing. The durable 18-month result demonstrated maintained 4-mm static two-point discrimination, which falls within the normal adult range of 2 to 5 mm for the fingertip pulp [12,13]. Nail growth was normal, and cold intolerance was absent. Only a mild persistent subjective sensory change was reported. These findings support true tissue survival and functional recovery.

This case suggests that perfusion assessment combined with mechanism of injury may be more predictive of salvage success than bridge size alone when evaluating distal fingertip injuries. The sharp cutting mechanism in this case likely preserved vessel architecture and minimized the zone of tissue injury, facilitating successful revascularization despite the minimal attachment and continued nicotine exposure.

## Conclusions

Primary reapproximation of a near-amputated Allen type II distal fingertip with minimal soft tissue attachment can lead to durable 18-month survival, preserved sensibility, normal nail growth, and excellent cosmetic outcome when perfusion is maintained, and the mechanism is a sharp cutting injury. Even with nicotine exposure, length preservation should be strongly considered when vascularity is evident and favorable prognostic factors, such as sharp mechanism and short ischemia time, are present.
